# The skin microbiome: impact of modern environments on skin ecology, barrier integrity, and systemic immune programming

**DOI:** 10.1186/s40413-017-0160-5

**Published:** 2017-08-22

**Authors:** Susan L. Prescott, Danica-Lea Larcombe, Alan C. Logan, Christina West, Wesley Burks, Luis Caraballo, Michael Levin, Eddie Van Etten, Pierre Horwitz, Anita Kozyrskyj, Dianne E Campbell

**Affiliations:** 1 School of Paediatrics and Child Health, University of Western Australia and Princess Margaret Hospital for Children, PO Box D184, Perth, WA 6001 Australia; 2 In-FLAME Global Network, of the World Universities Network (WUN), West New York, USA; 3 School of Science, Edith Cowan University, 270 Joondalup Drive, Joondalup, WA 6027 Australia; 4 Department of Clinical Sciences, Pediatrics, Umeå University, Umeå, Sweden; 5 University of North Carolina School of Medicine, Chapel Hill, North Carolina USA; 6 Institute for Immunological Research, University of Cartagena, Cartagena, Colombia; 7 Division of Paediatric Allergy, University of Cape Town, Cape Town, South Africa; 8 Department of Pediatrics, University of Alberta, Edmonton, Canada; 9 Children’s Hospital at Westmead, Sydney, Australia; 10 Discipline of Child and Adolescent Health, University of Sydney, Sydney, Australia

**Keywords:** Skin, Microbiome, Microbiota, Inflammation, Allergy, Cytokines, Biodiversity, Colonisation, Antibiotics, DOHaD, Ecosystems, Prevention, NCDs, Caesarean section, Pregnancy

## Abstract

Skin barrier structure and function is essential to human health. Hitherto unrecognized functions of epidermal keratinocytes show that the skin plays an important role in adapting whole-body physiology to changing environments, including the capacity to produce a wide variety of hormones, neurotransmitters and cytokine that can potentially influence whole-body states, and quite possibly, even emotions. Skin microbiota play an integral role in the maturation and homeostatic regulation of keratinocytes and host immune networks with systemic implications. As our primary interface with the external environment, the biodiversity of skin habitats is heavily influenced by the biodiversity of the ecosystems in which we reside. Thus, factors which alter the establishment and health of the skin microbiome have the potential to predispose to not only cutaneous disease, but also other inflammatory non-communicable diseases (NCDs). Indeed, disturbances of the stratum corneum have been noted in allergic diseases (eczema and food allergy), psoriasis, rosacea, acne vulgaris and with the skin aging process. The built environment, global biodiversity losses and declining nature relatedness are contributing to erosion of diversity at a micro-ecological level, including our own microbial habitats. This emphasises the importance of ecological perspectives in overcoming the factors that drive dysbiosis and the risk of inflammatory diseases across the life course.

## Background

As the primary interface with the external environment, skin ecosystem is home to complex yet still poorly understood microbial habitats and communities that reflect the health and diversity of the wider ecosystems in which we reside [1]. Resident microbes are increasingly viewed as an integral part of the functional unit of the skin and other body surfaces, interacting with tissues and immune networks to influence the health and function not only of local systems, but wellbeing more generally [1]. Indeed, the maturation and function of the systemic immune system in the young child is dependent on contact with microbes [2]. This, in turn, has implications for the development and function of virtually all organ systems, including the brain, which are profoundly influenced by the immune system. Locally, microbial-immune interactions in the skin are vital for optimal barrier function, pathogen defense, and tissue repair with the production of key anti-inflammatory and anti-microbial compounds to maintain healthy tissue homeostasis [3].

Just as in the gut, the metabolome in the skin reflects the combined functional metabolic activity of the microbes and our host tissues, and is greatly influenced by our environment and behaviour [4]. The very existence of this skin-environment interface raises important questions about how erosion of global biodiversity, and declining contact with the natural environments is affecting skin ecosystems and human health [5]. Examining this question in the context of the epidemic rise of allergy and other inflammatory diseases is informative because allergy is one of the earliest manifestations of inflammation often first observed in the skin as disruptions in barrier function and atopic eczema. Furthermore, the declining microbial diversity that has been long linked to the rise in allergic disease also has important implications for other organ systems across the life course [6].

### Roadmap to the current review

While there is much focus in the gut microbiota in this context, we argue that the role of skin ecosystems may be equally important, especially as defects in skin integrity, namely early onset eczema, have increased in tandem with modernity [7]. This highlights the importance of very early life events in the establishment of skin ecology and homeostasis and in pathways to disease. Moreover, it provides reason for optimism in the search for prevention, personalized medicine and effective strategies for treatment. Here in our narrative review, we first examine the structure and functions of the human skin barrier, as well as current knowledge concerning the microorganisms which play functionally essential metabolic roles in a particular cutaneous niche. Further, we describe the functional consequences of disturbances in the integrity of the cutaneous barrier, particularly as they relate to allergic diseases. This includes the mounting evidence which indicates there may be far-reaching, systemic immune repercussions, to local barrier disruptions. In exploring the environmental and lifestyle factors which help determine the interactions between the cutaneous microbiome and a healthy skin barrier, we take a broad view, discussing the total environment and the ways in which contact with large scale biodiversity might determine local skin micro-ecology. In a novel exploration, we expand upon discussions of farm exposure or pet ownership and open dialogue on the emerging construct of nature relatedness (individual affinity with the natural environment). This psychological asset which might play an underappreciated role in the links between environmental exposures and the microbiome. Finally, we look toward current and future possibilities whereby the skin microbiome might be leveraged for health promotion.

### The importance of the skin barrier

Despite volumes of research demonstrating otherwise, there is persistence of a long-held dogma that the stratum corneum – the outer layer of skin - is merely a collection of “dead” cells. In fact, the brick-and-mortar structure of the stratum corneum (SC) is highly biologically active and of major importance not only to skin health, but to overall health, throughout the life course. Disruptions of the epidermal barrier are well known in atopic dermatitis, psoriasis and rosacea; however loss of normal barrier structure and function is also of relevance in the most common skin condition - acne vulgaris - and for all humans as they proceed through the skin aging process [8, 9]. Since the SC represents an essential line of photoprotection, its breakdown in both pathology and through aging is also of relevance to the development of skin cancer over the life course [10, 11].

**Fig. 1:**
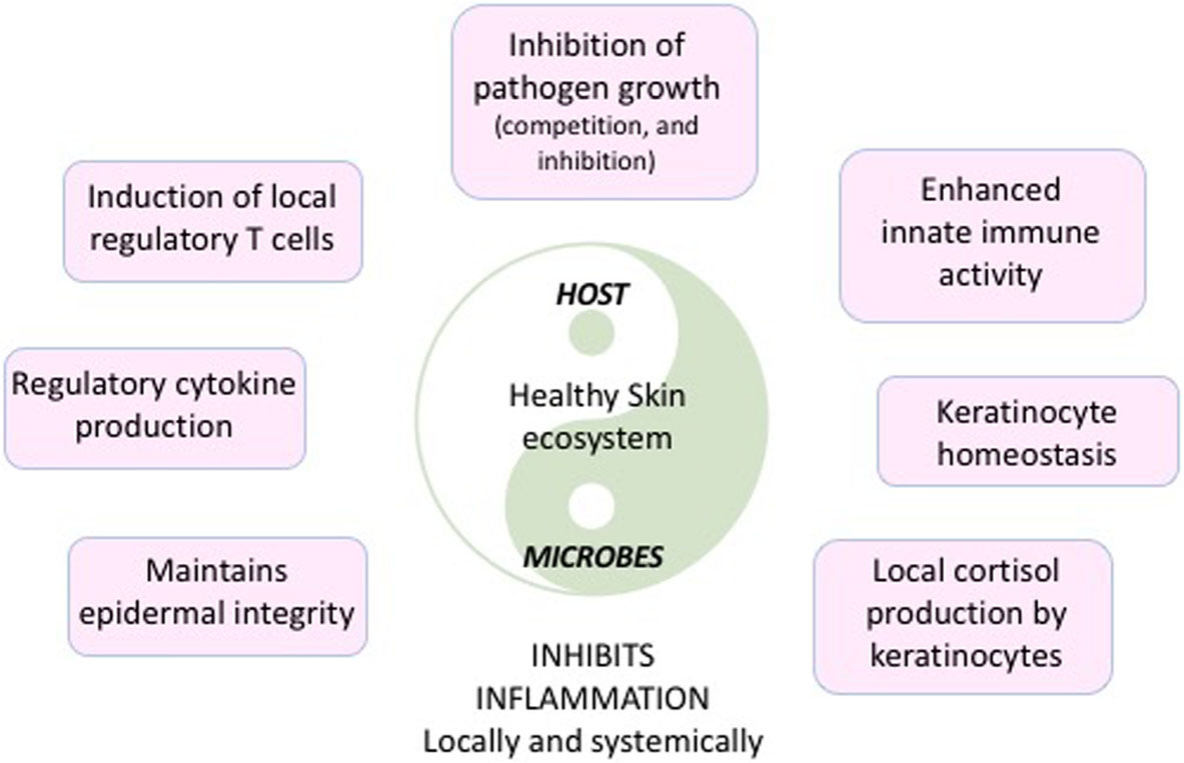
The interdependent mutualistic relationship between commensal microbes and the host maintains tissue homeostasis, inhibiting local inflammation. Regulatory responses generated in the skin also have systemic immunomodulatory effects

The SC consists of corneocytes that represent a tightly-organized set of bricks separated by a mortar of intercellular lamellar lipids (Fig. 1). The former are constructed of keratin macrofibrils and joined to one another by corneodesmosomes. The intercellular lipids are a collection of ceramides, cholesterol, and various fatty acids. Under normal circumstances this structure maintains an ideal level of skin hydration. However, the biological functions of the SC extend beyond hydration per se. The SC performs a variety of functions including, but not limited to: supporting the innate antioxidant system, production of antimicrobial peptides, activation of the host innate immune responses, and as mentioned, providing a line of defense against external threats from ultraviolet radiation and other environmental toxins, allergens, and pathogens [12].

Critical to our discussion, experimental research involving an ex vivo model shows that cutaneous microbes can influence the structure and function of the skin without penetrating the epidermis. Thus, microbes can set in motion the production of inflammatory cytokines within the outermost layer of the skin [13]. Once initiated, chronic inflammation can itself compromise the normal production of SC lipids [14], which means that cutaneous microbes may sit squarely within any discussions of the human epidermal barrier.

### The skin microbiome: The importance of cutaneous ecosystems

The human microbiome includes microorganisms and their collective genome residing in an anatomical niche. Remarkable advances in sequencing analysis such as bacterial 16S ribosomal RNA gene sequencing have allowed for tremendous insight into the previously obscure ecosystems operating on and within the human body. Indeed, given the functionally essential metabolic roles played by microbes and their symbiotic relationship with other forms of life, the holobiont perspective is one in which humans are a multi-species entity [15]. Bacteria residing on the skin mainly fall into four phyla: *Firmicutes*, *Bacteroidetes*, *Proteobacteria* and *Actinobacteria*. Within the bacterial groups, strain-level identification remains obscure. Since two different strains of the same bacterial species can have profound functional differences, there is a critical need to advance research in this more functional direction. Although less is known about other resident microorganisms such as viruses, fungi and parasites, they are likely to interact with the wider ecosystem and influence cutaneous immunity.

While the skin is arguably one complex ecosystem – it is made up of many different habitats and microbial communities. In any individual, the skin microbial composition is highly heterogeneous, and depends on the local microenvironment of the specific skin site. Studies of the topographical diversity across human body sites have found that moist versus dry skin areas “are likely as ecologically dissimilar as rain forests are to deserts” [16]. In general, these studies have identified three broad microenvironment types with characteristic microbial communities: sebaceous areas (where *Propionibacteria* species and *Staphylococci* species predominate), moist areas (where *Corynebacteria* species predominate, with *Staphylococci* also present) and dry areas (with mixed populations and greater prevalence of β-*Proteobacteria* and *Flavobacteriale*) [16]. Even then, the signatures vary with changes in the local microenvironment, between individuals and with health, behavior and environmental contacts (as discussed further below) [17]. It is important to recognize unique site-specific interactions that may not be captured with only a general perspective. Similarly, there are age-related differences, with a relative dominance of lactobacilli in neonatal skin versus propionibacteria in the mother [18].

**Fig. 2:**
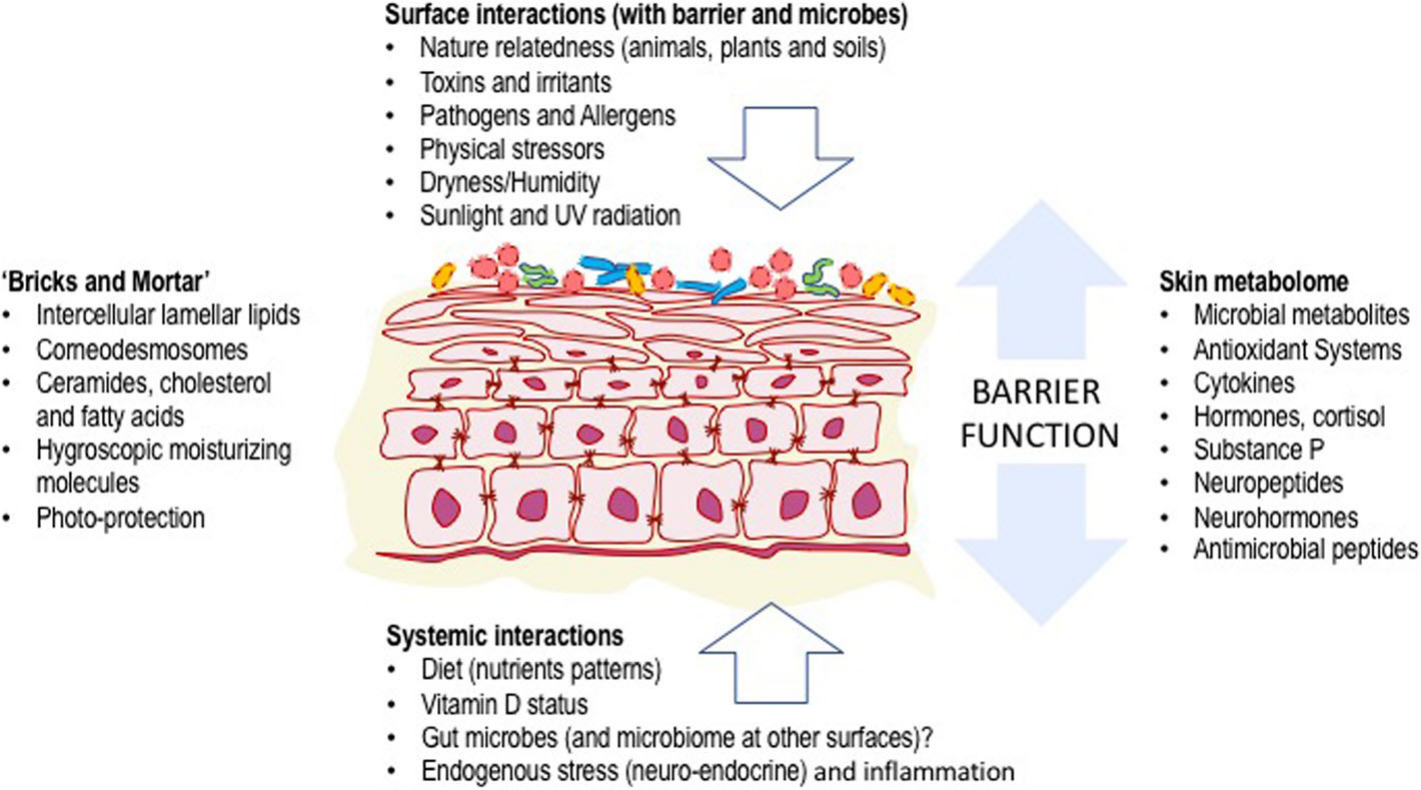
Both exogenous and endogenous factors interact with the physical and functional aspects of the skin barrier unit – through effects on both host cells and the skin microbiome – to alter both the integrity and the activity (hormonal, metabolic, and immune) of the skin

Of the Firmicutes, *Staphylococcus epidermidis* comprises more than 90% of all aerobic resident microbiota and has many mutualistic anti-inflammatory actions which promote barrier function and.

inhibit colonisation with potentially pathogenic strains of *Staphylococcus aureus* and potential pathogens [19]. This includes production of antibacterial peptides (bacteriocins), immunomodulatory properties (inhibition of inflammatory cytokine production) and enhanced expression of tight junction proteins. Many of these actions are mediated through activation of the innate immune receptors on keratinocytes and other local immune cells (via toll-like receptors) [20]. Thus, as with other organs, the skin innate immune system is a composite unit of interacting human and microbial elements and establishment of commensal microbiota is a key factor in developing initial homeostatic control of skin immunity (Fig. 2).

### Barrier disruption in allergic disease

Eczema is frequently the first manifestation of an allergic phenotype and clearly associated with epithelial barrier dysfunction, with increased transepidemal water loss (TEWL) a key feature of the disorder [21–23]. In fact, defective skin barrier integrity has been proposed as a primary and initiating event in the allergic phenotype [24], with allergen sensitization via skin giving rise to aberrant and dysregulated responses to innocuous environmental allergens [25]. Children with severe early-onset eczema have the greatest risk of IgE sensitization [26], with antigen transfer through a defective epidermal barrier a likely underlying mechanism [27]. Genetic contributions in eczema have been noted, including mutations of *FLG* which normally encodes for the protein profilaggrin, an essential structural component of the epidermal barrier [24, 28]. Although various gene mutations may contribute to dysfunctional epithelial development and mucosal integrity, they alone cannot account for the dramatic global increases in eczema and allergic disease [29].

There are recognized differences in the skin microbiota of individuals with established disease (reviewed in [19]) and at this juncture it is not clear to what degree this is secondary to skin pathology, or the extent to which skin dysbiosis also plays a role in the pathogenesis and propagation of disease. Recent twin studies indicate that the microbiome is a product of both genetics and the shared environment [30]. Indeed, strong associations have been found between the composition of skin microbiota and genetic factors related to skin barrier function. For example, *Corynebacterium jeikeium* abundance was lower in subjects containing the minor allele of *FLG* [31]. However, it is almost certain that skin microbes play a role in the initiation and amplification of inflammatory loops within the skin compartment [32].

*Staphylococcus aureus* colonisation and reduced microbial diversity is seen in over 90% of individuals with eczema compared with less than 5% of unaffected individuals (reviewed in [33]). *S. aureus* is seen in both lesional and non-lesional skin, as well as carriage in the nasal cavity [34, 35]. Genetic factors may predispose to nasal carriage (including glucocorticoid receptor gene polymorphisms) as well as environment factors such as exposure to biocides, including triclosan, which disrupt protective commensal ecology (reviewed in [19]). In particular, human colonisation with *S. aureus* in epidemiological studies has been associated with relative loss of mutualistic microbes particularly a subset of *S. epidermidis* which inhibits and destroys *S. aureus* biofilm formation by the production of serine proteases [36]. This provides perspective for observations that species of commensal staphylococci (including *S. epidermidis*) are reduced at two months of age pre-symptomatically in infants who subsequently develop eczema by one year of age [37]. A complex feedback loops suggested by the observation that improving barrier function and reducing skin inflammation significantly reduces *S. aureus* burden in children and adults with eczema. While the mechanisms and causal pathways are not yet defined, this nonetheless suggests a role for strategies that promote protective strains of *S. epidermidis* and other commensals*,* or at least prevent their loss. Importantly, the distinction between what is considered harmless or pathogenic lies not only in the inherent properties of the microbe, but in the health of the skin ecosystem, barrier integrity, and other inter-related local factors [20].

Recent studies using whole metagenome sequencing have identified distinct differences between baseline skin microbiomes of eczema prone subjects and normal healthy individuals, including microbiome signatures enriched for potential pathobionts of *Streptococcus, Gemella and Haemophilus* [38]. There were also functional shifts in microbiome-wide gene signatures associated with metabolic imbalance (primed to generate excess ammonia) providing a microbial explanation for dry alkaline pH changes associated with eczema flares [38]. These findings illustrate how the skin microbial communities, the surface microenvironment and the immune system cross-modulate each other to perpetuate inflammation.

### Links between the skin microbiome and systemic immune regulation

The skin manufactures and metabolizes steroid hormones, peptide neurohormones and neurotransmitters, including some which are further disseminated by sweat and sebum [39]. These chemicals make contact with cutaneous microbes and influence adhesion, growth and virulence. For example, experimental studies provide a pathway between psychological stress-induced increases in local Substance P production [40] and changes in skin microbiota [41, 42]. Increased Substance P is linked to eczema, acne and barrier dysfunction [43–45]. However, paradigm-shifting studies have allowed for an entirely different perspective, in which pathology is not entirely mediated in a unidirectional manner from brain to skin. New research places epidermal keratinocytes at the forefront of sensory systems, recognizing that they generate a variety of hormones and neurotransmitters that influence whole-body states and even emotions [46]. This includes not only the sensors of mechanical stress, temperature and chemical stimuli, but the capacity for glucocorticoid production via elements of the local hypothalamic-pituitary-adrenal (HPA) axis – acting as an independent steroidogenic organ [47]. Skin stressors including dryness and barrier disruption have been shown to stimulate cutaneous cortisol production, and this action may be mediated through activation of inflammatory cytokines such as IL-1β with systemic implications [48].

These hitherto unrecognized functions suggest that the skin plays an important role in adapting whole-body physiology to changing environments, and raises new questions about the impact of the local skin microbiome, acting by itself or interacting with gut microbiome [49], on these numerous systemic activities. As has been described for gut microbiome, the activities of the cutaneous microbiome are likely to extend far beyond local effects in the skin.

There is already clear evidence that both innate and adaptive immune function in the skin are influenced by the commensal skin microbiota [3, 50], including inhibition of pathogens, inflammation, immune development and homeostasis, repair and angiogenesis and T cell differentiation [51]. In studies involving germ-free mice, the absence of commensal skin bacteria compromises normal immune responses, most notably cytokine production [52]. Research using in vivo tissue fluorescence reveals a constitutive expression of tumor necrosis factor in the skin of healthy adult mice without signs of cutaneous inflammation. However, microbiota depletion eliminates this normal physiological function [53].

This microbiota-influenced local production of immune chemicals in the skin may have far-reaching *systemic* effects on immune regulation. This has been demonstrated in animal models, notably those examining the effects of environmental bacteria on allergic responses in distal organs such as the airways. It has been long recognized that exposure to rural environments rich in apathogenic bacteria, such as species of *Acinetobacter*, is associated with the inhibition of the development of allergic responses in humans. Notably, this association connects maternal exposure in pregnancy with reduced allergic risk in offspring [54]. Remarkably, *Acinetobacter lwoffii* isolated from these traditional farming environments (in rural Germany) and administered intra-nasally to pregnant animals can prevent the development of an asthmatic phenotype in the progeny [55]; a microbial induced Type 1 T helper (Th1) cell dependent effect mediated by epigenetic changes in the IFN gene [56].

**Fig. 3:**
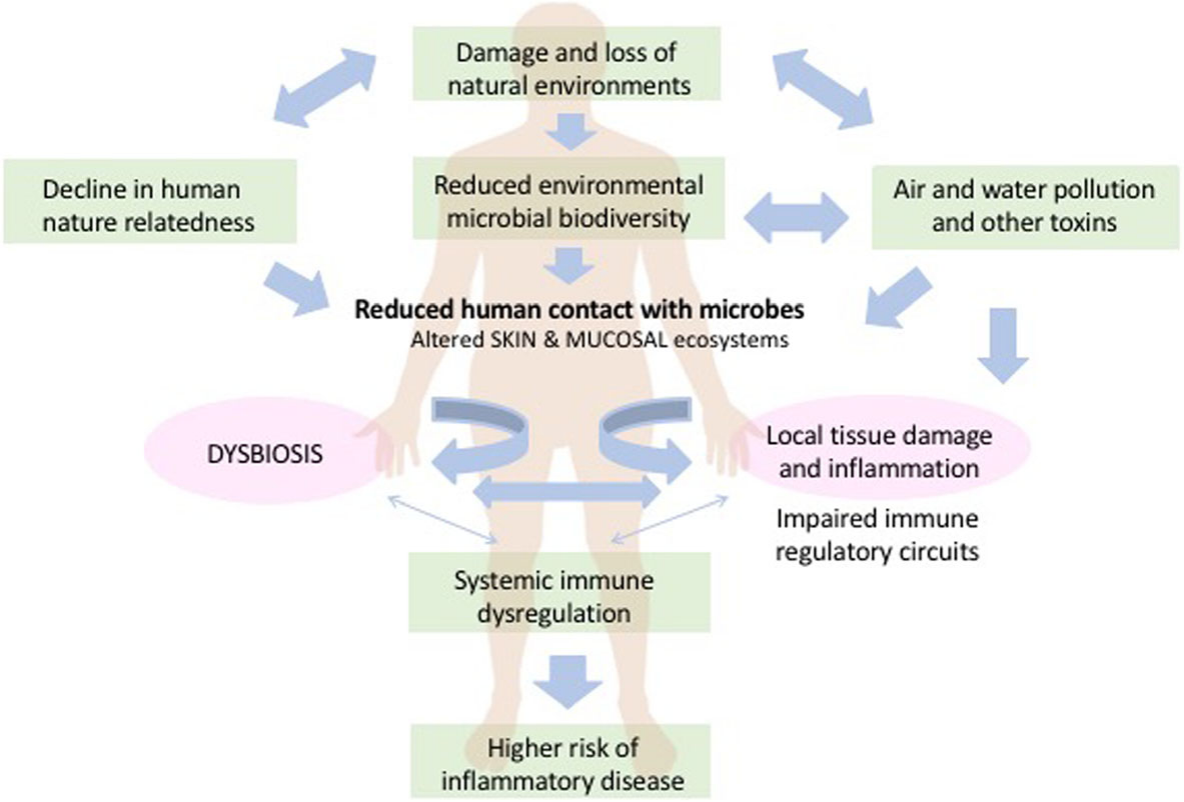
Erosion of environmental ecosystems is affecting biodiversity and microbial ecology. Together with declining nature-relatedness this is reducing human contact with immunomodulatory organisms found in natural environments – reflected in differences in skin microbes. This is increasingly being recognised as a risk factor for chronic inflammatory diseases

In the animal model, heat-killed *A*. *lwoffii* applied to the skin intradermally was shown to induce strong Th1 and anti-inflammatory and regulatory IL-10 responses locally in the skin, and that this then protected against *systemic* allergic sensitization and lung inflammation [57]. This is compelling evidence that skin commensals can play an important role in modulating systemic immune responses, including the propensity for systemic inflammation and has wider implications for other inflammatory NCDs (Fig. 3). It also provides another mechanism by which nature-relatedness (a person’s level of connectedness with the natural world [58]), biodiversity of ecosystems in which humans reside or to which they are exposed, access to greenspace and/or habitation in rural environments can have beneficial effects on physical and mental wellbeing [59].

In humans, the load of the gammaproteobacteria *Acinetobacter* on the skin of healthy teenage school children has been correlated with IL-10 expression in peripheral blood mononuclear cells [4]. Notably, atopic individuals have lower diversity of gammaproteobacteria on their skin, related to reduced environmental biodiversity of the home surroundings [4]. As IL-10 is an important regulatory cytokine this provides further evidence of an important link between the health and diversity of the environment at large and the health and diversity of human microbiomes, underscoring the significance of these interconnections for human health. Moreover, new research has linked remarkably low rates of allergy in asthma, allergic rhinitis and eczema in rural Karelia in North-Western Russia with cutaneous *Acinetobacter*. Insulated against westernization, this population maintains a traditional lifestyle in close contact with natural environments. Compared to residents of the geographically close (yet more westernized) Karelia region of neighboring Finland, rates of eczema are 10 times lower. Interestingly, the abundance and diversity of the *Acinteobacter* genus was much higher in Russian Karelia. Compared to Finnish children, the abundance of *Acinetobacter* was on average 3 times higher on the skin and 4 times higher on the nasal epithelium. There were also significantly higher levels of the aforementioned *A*. *lwoffii* on the skin [60].

Further studies are needed to investigate links with the risk of other NCDs, but it is noteworthy that animal models already illustrate connections between skin or oral contact with other non-pathogenic soil microbes, such as *Mycobacteria vaccae* and systemic regulatory immune function – and that this even has the capacity to influence the brain and behaviour [61, 62]. Many studies have shown that microbes, even when heat killed [63], have effects on innate immunity, and regulate gene expression in the brain [64], growth and glucose-insulin metabolism [65]. Just as studies have revealed unexpected links between the gut microbiota and organs such as the brain (reviewed in [66]), similar pathways from the skin are equally likely [67].

Functional loss, or even extinction [68] of ancient species from the human microbiome with progressive lifestyle change, raises important new dimensions in the relationship between the health of the environment and modern inflammatory diseases [59, 69]. This includes not only allergy and immune diseases, but many other conditions, including mental health, that are influenced by both the immune system and microbiome [66]. Original concepts of the ‘hygiene hypothesis’ have evolved more broadly into the ‘biodiversity hypothesis’, which recognizes that environments rich in diverse macrobiota and microbiota have a fundamental influence on the diversity of human microbial ecosystems. Human health is thus dependent upon biodiversity at the macro and micro scales [5]. The role of parasite-induced immunomodulation in maintaining immune system homeostasis and the consequences of its loss in modern societies is also increasingly recognized [70]. This provides greater impetus for more integrated, multisectoral approaches to environmental and human health [71].

### Early skin colonisation - developing immune tolerance to commensal skin bacteria

In animal models, tolerance to skin commensals such as *Staphylococcus epidermidis,*depends on neonatal exposure, mediated by a wave of activated regulatory T cells (Treg) rapidly entering skin [72]. Furthermore, there appears to be a critical developmental window [72], suggesting that the cutaneous microbiome composition in neonatal life is crucial in shaping adaptive immune responses to commensals, and that disrupting these interactions might have lasting health implications. Accordingly, murine studies show that dysbiosis and reduced diversity of skin microbes can influence the development of cutaneous inflammation and disease [72, 73]. While comparable human data are still limited, and cause vs. consequence (or both) is far from elucidated, there is evidence that alterations in the skin microbiome *predate* development of atopic dermatitis, with reduced abundance of commensal staphylococci in the antecubital fossa of infants later affected by disease [37]. This supports the protective role of some commensal microbiota. It also highlights the potential implications of perinatal practices which disrupt colonisation of the infant skin (and mucosal surfaces) including the use of antibiotics, soaps and disinfectants, delivery method, perturbations of maternal microbiota, and the general environmental context of the perinatal and early postnatal period (below).

It is likely, but not yet shown, that mucosal and skin integrity is specifically influenced by antenatal factors including the maternal microbiome and maternal environmental exposures. Maternally derived cytokines, allergens and other environmental agents are known to pass into amniotic fluid [74] and may influence developing mucosal surfaces and uncornified fetal skin- which may be more sensitive to such exposures [75]. Although contentious, it is possible that the feto-placental unit is not ‘sterile’ and that microbial colonisation of the foetus prior to birth [76, 77]. Indeed, metagenomic sequencing has revealed a rich placental microbiome in healthy pregnancies [78], of likely influence to developing metabolic and immune responses in the fetus. Certainly, there is evidence that an altered microbial composition during pregnancy may produce aberrant metabolites that adversely affect fetal development, including neurological outcomes [79] and cardiovascular development- with differences in infant aortic intima-media thickness [80]. These observations underscore the need to consider antenatal influences on fetal microbial colonization and immune development [81]. While there has been a dominant focus on how this may modulate gut colonisation, it is not clear how this is related to the subsequent skin ecosystem.

The maternal microbiome may play an important role in initiating foetal immune tolerance to commensal microbiota and therefore promoting optimal postnatal colonisation. Potentially, factors which induce low-grade inflammation in utero could influence or damage tight junctional proteins that maintain skin and mucosal integrity. The resulting abnormalities of epithelial development may predispose to very early mucosal immune dysregulation associated with the appearance of clinical food allergies and eczema shortly after birth [82]. It also remains to be seen how antenatal mucosal disturbances interfere with postnatal colonisation.

After birth, there is large-scale acquisition of skin microbes, and the composition of this complex ecosystem is influenced by a myriad of perinatal factors including mode of delivery (vaginal or caesarean), antibiotics, and a range of maternal and environmental factors [33, 83, 84]. Early infant skin colonisation is also modulated by the natural antimicrobial properties of vernix caseosa, the protective biofilm covering the skin of the fetus during the last trimester of pregnancy. This favors colonization by skin commensal microbes over pathogens [50, 51, 84]. The anti-oxidant and wound healing effects of vernix also protect barrier integrity (reviewed in [84]). Thus, “routine” post-delivery washing of newborns with soaps and/or detergents is likely to influence both patterns of colonization and skin barrier function. Skin colonisation appears to evolve in complexity over the first years of life and remains relatively unstable until early adulthood [84, 85].

Compared to the gut, the skin microbiome appears to have more variability over time [33] and displays wide variability between individuals [3, 86]. Factors that modulate the health and diversity of these maturing ecosystems have significant potential to modulate local and systemic immune networks and thus influence predisposition to disease, with ongoing bi-directional interactions between host and colonising microbes. The Human Microbiome Project is continuing to examine these complex relationships and it is hoped it will reveal important associations between microbial signatures (of the skin and other ecosystems) and disease predisposition for novel therapeutic targets [3, 86].

### Factors which can influence skin colonisation and the subsequent ecology

**Fig. 4:**
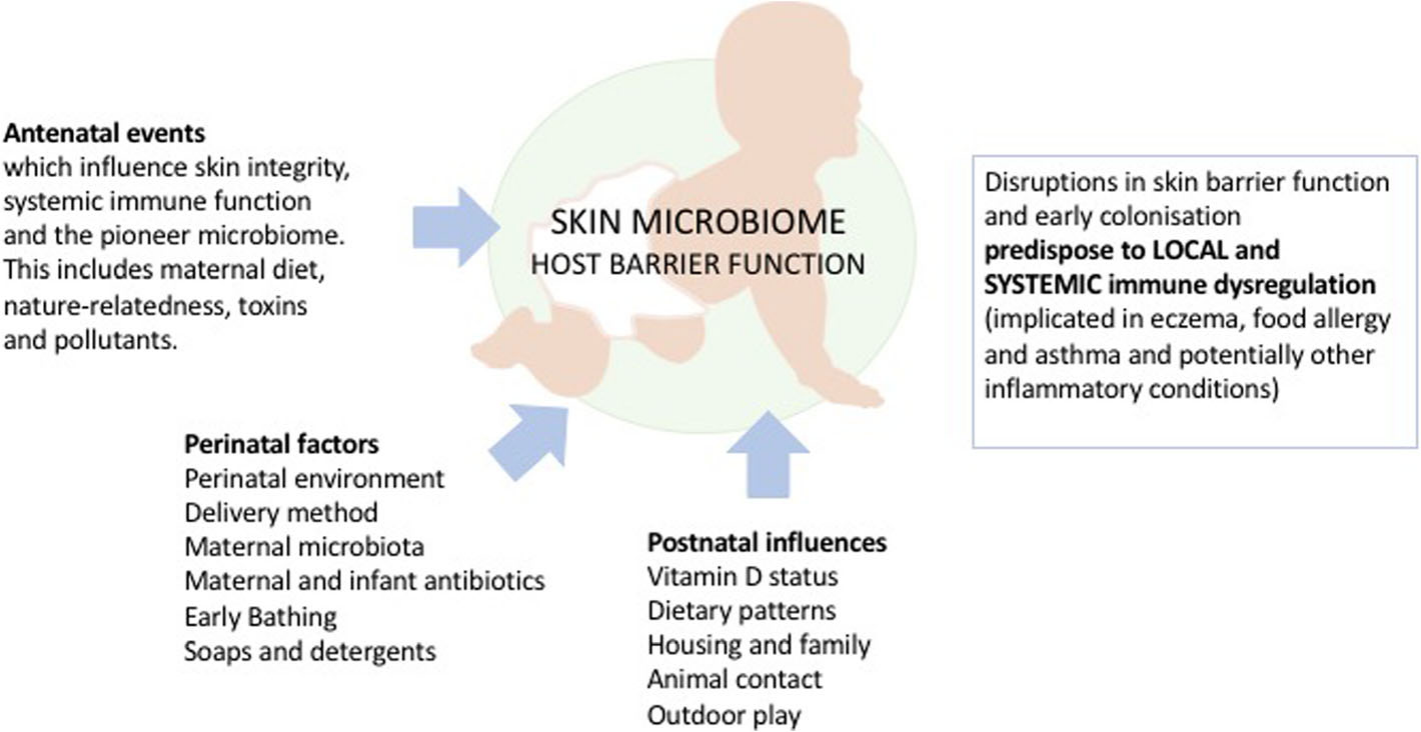
Early life is a critical period for establishment of both the microbiome and immune responses, with long term implications for health. Understanding modulating factors during this period could lead to targets for disease prevention

In essence, everything that people touch, bathe in, breathe, eat, and drink is reflected in their many microbial ecosystems, including the skin. This underscores the important influence of environmental conditions on colonizing microbiota at every age, but particularly in early life when various surface ecosystems and immune pathways are being established (Fig. 4). For this reason, many factors that influence the maternal microbiota will be relevant to the establishment of fetal and infant colonisation, including nutrition, psychological stress and medication (particularly antibiotics and biocides) [87–89]. Infant skin colonization is also affected by maternal hormonal influences of pregnancy which are known to alter the cutaneous environment, sebum production, although these effects are transient. Delivery method has a major influence on initial colonizing bacteria in all habitats including the infant skin [83]. Unlike vaginally delivered infants, who are colonized by bacteria from the vaginal community, infants born by C-section are dominantly colonized by *Staphylococcus* and other taxa reflecting maternal skin flora [83, 90]. While the effect of delivery method on infant gut flora persist over the first 3–6 months of life [91]during critical windows of immune development, the longitudinal impact on skin colonisation patterns is less clear.

While the effects of antibiotics and dietary practices are well studied for the gut microbiome, the effects on the skin, especially during early life are not clear. In one recent study, feeding method did not have a significant effect on the skin colonisation patterns [37]. Further studies are needed to document how the microbial ecology of the skin becomes established and stabilizes over the first years of life, and how variations influence immune development and disease risk. It is likely that the ambient environment has an important effect on the developing skin microbiota, including contact with detergents and hygienic products, soaps, moisturizers and cosmetics. The timing and frequency of infant bathing after delivery is also likely to be important. We speculate that excessive washing with detergents, particularly in infants at risk of eczema, impairs skin barrier function and alters skin colonization thereby increasing the risk of developing eczema and sensitisation, but further studies are needed to examine this. A protective effect of early and frequent skin moisturizing of infants at risk of eczema has been shown in a small clinical trial [92], however the composition of the microbiome was not reported. There is also suggestive data that modulating the gut flora with probiotics in infants with eczema can modulate systemic immune responses [93], and improve local skin disease [94]. Although the mechanisms are not clear, it is possible that this could be mediated through systemic modulation of both local immune responses and the cutaneous microbiome.

Family and household contacts also have an important influence. Closer similarities of skin commensal bacteria between family members residing in the same home than individuals from different households has been reported [95]. Pets are a major source of household bacteria shedding hair, dander, saliva, faecal particulates, and carrying soil micro-organisms. The same study showed that pet ownership had an important effect – with dog owners sharing more skin microbes with their dogs, and with other household members [95]. This reflects differences in the biodiversity of household dust samples depending on whether families have pets, or have children who attend daycare [96]. New studies also show that individuals have a unique personal microbial ‘cloud’, emitting upwards of 10 [6] biological particles per hour, and influencing the surrounding environment [97]. People also transfer microbial communities across indoor surfaces [98], and the microbiome signatures of devices such as mobile phones have been shown to closely reflect that of their owners [99]. Other factors including the ambient temperature, ventilation, co-occupancy, humidity, environmental contact, air quality and sunlight (ultraviolet) light are also likely to contribute to individual and geographic variation in skin colonisation [33, 100]. Differences in skin disease profile in tropical areas, including reduced prevalence of eczema than temperate countries [101], suggests that comparative geographical studies of skin microbiota could identify specific protective factors that might be translated for therapeutic purposes.

The skin microbiome appears to have greater variability over time than the gut [33], suggesting that this may be modified more readily by environmental exposures. This has been shown with personal contact (even in sporting activities), or changes in environment, such as Antarctic expeditions and even space travel by astronauts [102–104]. It also suggests the potential for modifying intervention as preventive or therapeutic avenues.

### Nature relatedness and environmental biodiversity as a major factor in human microbial diversity

The biodiversity of the human microbiome is, to a considerable extent, a function of the macrobiome– the ecological health and the biodiversity of the surrounding environment – and our interaction with it [5]. At the macro scale, diverse and complex ecosystems are inherently more resilient to threats and fluctuations. It may be possible to apply ecological theory to understand how losses in diversity at the micro scale - within human microbial ecosystems - may also represent a threat to health. Although there are exceptions [105, 106], the preponderance of evidence suggests that overall diversity of microbes in a select human niche equates, broadly, to health [107]. This raises many, as yet unanswered questions about the impact of biodiversity loss on human health and the spiraling modern burden of NCDs- as ‘green space’ is progressively displaced by ‘grey space’ along a gradient of urbanization [108, 109].There is now consistent evidence that environmental degradation, whether by climate change, invasive species or industrial activity, is linked to diminished human physical and mental health [110, 111].

While the mechanisms are complex and multifaceted [112], the microbial biodiversity encountered through contact with natural environments, especially early in life, may be one critical factor in the reported health benefits of nature relatedness [113]. There are now a number of studies correlating validated measures of nature relatedness (NR) with health benefits [58, 114, 115]. These NR scales assess personal interconnectedness with nature on multiple levels, including cognitive, affective, and physical connections. Higher scores on NR scales are associated with spending time within outdoor natural environments [116].

While this is multifactorial, higher microbial exposure in traditional environments is likely to be an important component of the disease protective effects. For example, contact with microbial endotoxin is a major factor implicated in the reduced rates of asthma and allergic disease in traditional farming communities in both Europe and North America [117–119]. Saprophytic bacteria (from soil and vegetation) are increasingly recognized for their immunomodulatory effects, and the separation from these evolutionary relationships is of growing concern [120].

The very high taxonomic diversity of hunter-gatherer microbiomes, such as the Hadza tribe of the East African Rift Valley [121], provide a snapshot of what has been eroded in urban western populations where exposures to such bacterial assemblages have diminished. Individuals who maintain traditional non-westernized lifestyles have a higher frequency of soil microbes found on their hands [122]. Differences in cutaneous microbiota of rural compared with urban adults [123] suggest that contemporary cities and lifestyle behaviours are separating humans from microbialexposures in the external environment with which they evolved [108]. As noted in a World Allergy Organization Consensus Statement: “*Biodiversity loss leads to reduced interaction between environmental and human microbiotas. This in turn may lead to immune dysfunction and impaired tolerance mechanisms in humans*” [124].The effects of biodiversity on immune health have implications for not only allergy [124]and autoimmune disease [125], but many inflammatory NCDs, including mental health disorders [59, 126].

The indoor microbiome in house dust samples reflects the external environmental and house dust from urban homes is relatively less diverse in microbial components compared to dust in rural homes [127]. Skin Proteobacteria are more frequent in people living near agricultural and forest environments [5]. Even within urban settings the amount of ‘green space’ and biodiversity in the vegetation surrounding the primary residence is an important determinant of commensal skin bacteria [4, 120]. Soil microbial communities in city parks are different from forests and are shaped by vegetation type and the age of the park [128]. In a study of four cohorts comprising 1044 children from Finland and Estonia, closer contact with forest and agricultural land (within 2–5 km of the home) was associated with significantly reduced risk of allergic sensitization, and early exposure to ‘green’ environments was especially protective [120]. Moreover, the authors observed that land use and environmental biodiversity contributed to 20% of the variance in relative abundance of Proteobacteria on the skin of healthy individuals. This is also consistent with previous studies showing the protective effect of early-life exposure to rural environments and animals against the development of asthma and allergies [85, 86, 129]. Although, this link has not been causally proven in humans, collectively these data support the hypothesis of a strong environmental effect on the potentially immunogenic commensal microbiota derived from natural environments, and that cutaneous contact is an important pathway.

Even the microbiome signatures of the air people breathe are different in ‘green’ versus ‘grey’ environments [130].Vegetation makes a significant contribution to the airborne microbial content – up to 10-fold higher than nearby non-vegetated built areas [131]. Air sampling studies have shown that airborne bacterial communities from parks are different from parking lots, with the proportion of vegetated area within a 50 m radius of sampling stations explaining 15% of microbial composition variation [130]. This illustrates the extent of potential interaction between the personal human microbiome ‘cloud’ and the ambient environment, not merely through contact with plants and soils, pets, water and food, but even the surrounding air.

Since the dermis is not a complete barrier, but rather a filter to microbial access to deeper dermal stroma [132], it is not surprising that cutaneous microbes derived from soils, air and plants can influence systemic immune function. As noted above, the level of skin Proteobacteria in teenagers is positively correlated with baseline expression of anti-inflammatory IL-10 by peripheral blood mononuclear cells [4], and similar organisms conferred protection against allergic responses in mice [57]. Aside from the effects of skin Proteobacteria (discussed above), other non-pathogenic microbes (*M.vaccae)* found in soil and water have been shown to improve cognition, anxiety and have other neurological benefits in rodent models when added to food [133, 134] or administered through the skin (subcutaneous heat-inactivated *M. vaccae*) [135]. Potential mechanisms include suppression of inflammatory responses and upregulation of CD4 + CD25 + Foxp3+ regulatory T cells and suggest that the immune system is a central pathway mediating the effects of nature on health [108].

With this background we can turn toward a practical way in which NR can confound research. Although a fairly robust body of research links domestic dog ownership with reduced risk of eczema in children [136, 137], the mechanisms behind this relationship are obscure. As mentioned above, dog ownership is associated with co-sharing cutaneous microbes with the pet. However, NR is associated with pet ownership [58]. Thus, if NR itself (and/or other psychological attributes) drives toward the desire to cohabitate with a dog in the first place, it may also be associated with a set of lifestyle factors – spending time outdoors, physical activity, stress reduction - that might push toward unique, and protective, microbial assets. The links between dog ownership, the human microbiome and immune functioning are extremely complex; they cannot be separated from the larger ecosystem and the biopsychosocial context.

### Strategies to promote the health and integrity of the skin and its microbial communities as potential pathways to preventing disease

Promoting optimal establishment of human microbial communities for long term health will ultimately depend on approaches at both the societal and individual level. Unless the greater adverse forces affecting the ecology of the wider urban environment are addressed, the benefits of individual strategies may be limited. On the larger scale, social determinants of health extend beyond the well-recognised lifestyle risk factors (diet, exercise) for disease, to the ecological determinants of health [138].This calls for integrated approaches that span all aspects of urban design and city planning, and which encourage human interaction with nature, plants, soils and clean air – making cities into microbe-friendly environments [139]. Environmental remediation and restoring ‘green’ space in urban blight can have many health benefits (reviewed in [59]).This can be achieved in aesthetic ways that also promote biodiversity [140]. Importantly, relatively simple transformation of vacant urban lots with trees and other forms of vegetation can have wider physical, mental and social benefits [141, 142]. Strategies to improve the quality and diversity of urban green spaces and plant communities will improve the health of urban systems and urban microbiomes, including those of humans [4, 128].

But better access to ‘green space’ is only part of the story. Healthier ‘living’ buildings are also required to encourage the growth of healthy ecosystem through the use of bioreceptive materials with textures and pH that encourage microbes and plants to grow naturally [143]. Achieving these goals will depend on increasing the health of the ‘external’ environment and ‘bringing the outside in’ – with opportunities for introducing and/or cultivating benign microbiota in the built environment. The quest for research indicators supportive of ‘evidence-based design’ for health promotion in the modern environment will require closer collaboration between the medical and health sciences, urban planners and ecologists.

With shifting perspectives around human interdependence on natural and microbial ecosystems, there are ongoing concerns about the overuse of biocides and biostatic agents. Although aimed at sterilizing surfaces and substances, it is unclear whether the public overuse of such agents provides new niches for resistant opportunistic microbes, ultimately representing a threat to human health. This is increasingly seen in clinical settings where unwell patients may be more vulnerable, and is of particular relevance in the neonatal setting. While reducing the threat of infection during the perinatal period is arguably the most significant achievement in reducing maternal and infant mortality, there is little doubt that antibiotics and biocides have had unintended consequences. Strategies to improve maternal and neonatal colonisation are now increasingly explored to promote optimal metabolic and immune health. It is now recognised that protecting the vulnerable developing ecosystems of the neonate, especially in preterm infants, is not best served by antibiotics alone with prebiotics and probiotics now routinely used to reduce the risk of life-threatening conditions such as necrotising enterocolitis in many centres [144–146].

Aside from infant bathing practices (discussed above), early microbial intervention may be particularly relevant for infants delivered by C-section, who are otherwise deficient in the normal vaginal inoculum [83]. Practices, such as seeding the skin of C-section infants by swabbing the infants mouth, face and body with maternal vaginal microbes [84] can partially restore cutaneous and mucosal microbiota of these infants [90], and are increasingly commonly performed. In this context, it is critical to exclude maternal carriage of pathogens such as group B *Streptococcus* (GBS) and further studies are needed to examine the longer-term effects.

Akin to faecal transplants for gastrointestinal health, researchers have experimented with skin microbiota transfer. Transplanting microbiota from the forearm to forehead demonstrates relatively rapid assimilation to the native microbial profile at transplanted site (forehead), indicating that environmental characteristics play a strong role in shaping skin bacterial communities at sebaceous sites such as the forehead [147]. However, this does not preclude the transfer of healthy donor microbes from and to similar anatomical locations where dysbiosis may be a factor in lesions. Indeed, new research in an animal model of atopic dermatitis shows that the transfer of live microbes derived from healthy human cutaneous tissue can enhance barrier function, modulate innate immunity activation, and control the overgrowth of *S. aureus* that typically accompanies skin inflammation [148]. Such research not only points to dysbiosis as a causative factor, it also opens the door of therapeutic possibility for the use of healthy donor microbiota in many skin pathologies.

Along the same lines, emerging research suggests promise for the topical delivery of specific live microorganisms, microbial lysates, and/or prebiotic substrates that selectively promote cutaneous microbial growth [149–151]. Although the research supporting such applications remains limited, there is evidence that the topical applications of probiotics can enhance local barrier function and immune responses at the site of application (reviewed in [152]). There are also preliminary studies indicating that emollients supplemented with nonpathogenic bacteria (*Vitreoscilla filiformis*) can regulate the skin microbiome, restore the barrier function and reduce eczema flares [153, 154]. Moreover, prebiotic fiber such as glucomannan has been the subject of recent experimental and clinical work; in vitro research suggest prebiotics might selectively inhibit pathogenic bacteria [155], while a small clinical trial showed that topical application of glucomannan at 5% improved acne lesions [156].

While far from unequivocal, there is evidence from human intervention studies that *orally* ingested prebiotics and probiotics can have systemic immune benefits that may be manifest in the *skin*. These benefits may extend to a reduced risk of allergic disease in early life (reviewed in [157, 158]). In a recent clinical trial inclusive of skin biopsy, oral probiotics were shown to influence gene expression of cutaneous IGF-1 and forkhead box protein O1 (FOXO1), both of which can regulate skin inflammation and local repair processes [159]. Preclinical studies show that orally ingested probiotics can positively influence the integrity of the stratum corneum, reduce the generation of radical oxygen species and prevent TEWL under the stress of ultraviolet radiation [160].

More than a decade has passed since the use of oral probiotics was hypothesized to lower fatigue and depressive symptoms [161, 162]. While the overall pool of studies remains small, meta-analyses of published intervention studies supports these original ideas; probiotics have been shown to improve emotional outlook and anxiety [163, 164]. Thus, oral bacteriotherapy might improve the skin barrier via psychoneuroimmunological pathways [66]. Studies indicate that orally consumed probiotics can influence the nasal microbiome [165], and manipulation of gut microbes can shift the lung microbiome [166]. However, it is remarkable that amongst volumes of preclinical research and multiple human intervention studies demonstrating benefit of oral probiotics in atopic dermatitis (and other skin conditions where the barrier may be compromised), there has been no attention paid to whether the success with oral bacteriotherapy is mediated by alterations to the cutaneous microbiome. An urgent question remains: are oral probiotics influencing skin microbes?

Finally, while commercial interests are slanted towards product development, it is critical to emphasise the importance of holistic measures which promote a healthy relationship with environments and ecosystems – especially in very young children. From a scientific perspective, the connections between experience in outdoor natural environments, exposure to microbial biodiversity, and enduring aspects of health require further study. However, there are multiple, collateral benefits associated with outdoor play in overall childhood health and development. More balanced indoor (screen) time with increased outdoor play time has overlapping benefits on sleep [167], academic achievement [168] and mental health [169]. Childhood experience of nature also builds emotional affinity for natural environments and the motivation to protect biodiversity in adulthood [170–173] and thereby improve the chance of ‘paying forward’ ecosystem health for generations to come.

### Metagenomic elements and clinical ecology

The available evidence suggests that the skin will follow in the same direction as the more robust body of research concerning the gut microbiome – that is, a high degree of individuality and strain-level differences in the microbiome that will likely be the ‘fingerprint’ of the presence or absence of disease. As mentioned earlier, the major limitation of the accumulated microbiome research has been lack of identification below phyla and species level. However, examination of the gut microbiome at the strain level has revealed remarkable functional differences within the same species [174].

Recent investigations involving the skin microbiome suggest a similar species-level fingerprint. Metagenomic shotgun sequencing is overcoming the lack of molecular insight provided by phylogenetic marker-based sequencing. New research involving patients with acne provides an elegant example of clinically-relevant knowledge provided by ultra-deep sequencing. Historically, *Propionibacterium acnes* has been considered a causative agent in acne. With only limited success [175], treatment approaches are often driven toward complete elimination of this species from the follicle. However, a closer look using metagenomic shotgun sequencing reveals that a high percentage of *Propionibacterium* in general, and *P. acnes* phage in particular, is characteristic of healthy skin. At the species and strain level acne patients had a greater diversity of *P. acnes* with enriched virulence-associated factors and *reduced* abundance of metabolic synthesis genes [176].

The results of this new study suggest that future bacteriotherapy will transcend the ‘cannonball’ targeting of an entire genus and work toward supporting the growth of beneficial cutaneous microbial strains, while at the same time attempting to selectively limit the growth of barrier-disturbing, disease-causing strains. In order to achieve this aim, the most obvious approaches will be very selective agents. However, future therapeutics might also address the *entire* microbial ecosystem in a holistic manner. In other words, solutions might be found by asking ‘*what are the upstream causative factors that shift entire microbial communities?*’, changes that in turn, allow for the emergence of virulent strains within the community. Although our focus is on the skin microbiome and increasing rates of allergic skin diseases, acne is no less a disease of westernization [177, 178].

## Conclusions

The skin ecosystem and its many commensal communities comprise a functional sensory unit which produces previously unrecognized systemic signals through both keratinocytes, specialised antigen presenting cells and the cutaneous immune networks. The recent discovery that the skin is an independent steroidogenic organ, with the capacity to influence whole-body states and emotions [46, 47], provides new perspective to the central importance of microbial communities and cutaneous homeostasis. Abnormal skin colonisation may contribute to abnormalities of epithelial development, integrity and predispose to local and systemic immune dysregulation – often first manifest as food allergy and eczema. Understanding these pathways may lead to therapeutic approaches to not only prevent and treat inflammatory skin disease, but other systemic conditions. The importance of the early environment in the life-long risk of NCDs also underscores the need to take a developmental approach to optimising colonising ecosystems.

The fundamental influence of the health of ecosystems in which humans live, on the diversity of human skin and mucosal microbiota, underscores the relevance of climate change, rapid urbanization, environmental degradation and gross biodiversity loss to human health, including the modern disconnection from nature [59]. We cannot truly promote health without widening our view to the wider dysbiosis of Earth’s ecosystems [109] and the reality that NCDs will be increasingly driven by loss of, or degradation of, these ecosystems at the macrobiological and microbiological levels. As we learn more about the microbiome it is obvious that personalized medicine must move toward a new clinical ecology, in which the external world (of our lifestyle within and around our habitat) matters to the ecosystems of our skin, intestinal and other personal habitats [59].

The evolutionary-rooted relationships with our microbiota are mediated at the immune interface and intertwined with the global burden of NCDs. Thus, there is a need to explore microbial solutions in early life through nature contact, controlled seeding of microbes and clever urban environmental design [61, 90, 179]. In order to accomplish this, a better understanding of interconnected ecology of humans, microbes and the environment is required. This does not necessarily mean forsaking technology and modern conveniences to return ‘back to nature’, it nonetheless means striking a new balance for ecological justice which ensures more equitable access to healthy natural environments, fresh healthy, minimally-processed foods and healthy urban systems [59]. By moving ‘forward with nature’, working with microbes on the skin and countless other niches, we can support a terrain of health. With this approach we may have the best chance of improving both human and environmental health for future generations.

## Abbreviations

DOHaD: Developmental origins of health and disease
FOXO1: Forkhead box protein O1
GBS: Group B *Streptococcus*
HPA axis: Hypothalamic-pituitary-adrenal axis
IGF: Insulin-like growth factor
NCDs: Non-communicable diseases
NR: Nature relatedness
SC: Stratum corneum
TEWL: Transepidemal water loss
Th1: Type 1 T helper cell
Treg: Regulatory T cells
